# Psoriasis and Chronic Viral Infections (HIV, HBV, and HCV): Therapeutic Approach and Safety

**DOI:** 10.3390/medicina62091796

**Published:** 2026-09-18

**Authors:** Gustavo Almeida-Silva, Diogo Couto-Sousa, Inês Tribolet-Abreu, Filipe Monteiro, João Ferreira, Joana Antunes, Paulo Filipe

**Affiliations:** 1 Department of Dermatology, Hospital de Santa Maria, Unidade Local de Saúde Santa Maria, Avenida Prof. Egas Moniz, 1649-035 Lisbon, Portugal29722@chln.min-saude.pt (F.M.); 18636@chln.min-saude.pt (J.A.); 10717@chln.min-saude.pt or paulolealfilipe@gmail.com (P.F.); 2 Dermatology University Clinic, Faculty of Medicine, University of Lisbon, 1649-004 Lisbon, Portugal; hjoao@medicina.ulisboa.pt; 3 Gastroenterology and Hepatology Department, Hospital de Santa Maria, Unidade Local de Saúde Santa Maria, 1649-035 Lisbon, Portugal; diogocoutosousa@gmail.com; 4 Gastroenterology and Hepatology University Clinic, Faculty of Medicine, University of Lisbon, 1649-004 Lisbon, Portugal

**Keywords:** psoriasis, HIV, hepatitis B, hepatitis C, biologics

## Abstract

*Background and Objectives*: Psoriasis is a chronic immune-mediated inflammatory disease that often requires long-term systemic therapy. Coexisting human immunodeficiency virus (HIV), hepatitis B virus (HBV), or hepatitis C virus (HCV) infection complicates treatment selection because clinicians must balance control of skin and joint inflammation against risks of opportunistic infection, viral replication or reactivation, hepatotoxicity, and drug interactions. *Materials and Methods*: This narrative review summarizes evidence from guidelines, systematic reviews, cohort studies, case series, expert recommendations, and regulatory sources. *Results*: In patients with suppressed HIV RNA, stable or reconstituted CD4 counts, no active opportunistic infection, and reliable follow-up, selected systemic agents, including biologics, may be considered after individualized risk assessment. In HBV infection, reactivation prevention through antiviral prophylaxis or close monitoring is central. In HCV infection, fibrosis stage, active hepatitis, drug interactions, and timing of direct-acting antivirals should guide management. *Conclusions*: Chronic viral infection should not be regarded as an absolute barrier to modern psoriasis therapy.

## 1. Introduction

Psoriasis is a chronic systemic inflammatory disease affecting the skin, nails, and joints. Its immunopathogenesis is dominated by dendritic-cell activation, IL-23-driven Th17 differentiation, TNF-alpha, IL-17A/F, IL-22, and downstream keratinocyte amplification loops [1]. Moderate-to-severe disease frequently requires systemic therapy, including methotrexate, cyclosporine, acitretin, fumarates, apremilast, TYK2 inhibition, biologic agents targeting TNF-alpha, IL-12/23, IL-17, and IL-23, and, more recently, oral IL-23 receptor antagonism [2,3,4,5,6,7,8].

The coexistence of psoriasis with HIV, HBV, or HCV infection creates a clinically important therapeutic dilemma. Although these viruses differ substantially in their pathogenesis and clinical consequences, each can modify the risk-benefit balance of systemic psoriasis therapy through effects on immune competence, viral replication or reactivation, hepatic reserve, and pharmacological interactions. HIV may be associated with severe, erythrodermic, palmoplantar, inverse, rupioid, sebopsoriasis-like, or treatment-refractory psoriasis [9,10,11]; HBV can reactivate when immune control is impaired by immunosuppressive or biologic therapy [12,13,14,15,16,17]; and active HCV infection or advanced fibrosis heightens concern for hepatotoxicity and drug interactions [18,19,20,21,22]. Thus, chronic viral infection modifies treatment selection not by imposing a uniform contraindication, but by requiring pathogen-specific assessment of infectious, hepatic, and pharmacological risk.

Modern antiviral therapy has substantially changed this therapeutic landscape: ART can suppress HIV replication and restore immune reserve; entecavir, tenofovir disoproxil fumarate, and tenofovir alafenamide can control HBV; and modern DAA regimens cure most HCV infections [12,13,14,15,18,19,20]. In parallel, psoriasis treatments have become more targeted. The key clinical question is therefore no longer whether systemic therapy is categorically contraindicated, but how it should be timed, selected, and monitored. Therapeutic decision-making should integrate HIV RNA and CD4 count when applicable, HBV or HCV viral activity, liver function and fibrosis stage, potential drug–drug interactions, psoriasis severity and urgency, psoriatic arthritis, comorbidities, and patient preferences [21,22,23,24]. Accordingly, this review synthesizes the pathophysiological and clinical evidence linking psoriasis with HIV, HBV, and HCV and proposes a practical risk-adapted framework for systemic treatment.

## 2. Pathophysiological Links Between Psoriasis, HIV, and Viral Hepatitis

### 2.1. Psoriasis and HIV

The relationship between HIV and psoriasis is paradoxical: psoriasis is classically considered T-cell mediated, yet it may worsen as CD4 counts fall [9,10,11]. This apparent contradiction likely reflects persistent immune dysregulation rather than a simple dependence on absolute CD4-cell numbers, with altered CD4/CD8 balance and cytokine-driven inflammatory pathways sustaining psoriasiform inflammation despite quantitative CD4 depletion [9,10,11].

The review by Alpalhão, Borges-Costa, and Filipe emphasized several clinically useful points: psoriasis in HIV-positive individuals may have higher incidence, atypical and more exuberant manifestations, and frequent treatment recalcitrance; at the same time, therapeutic choices have historically been constrained by concern for severe immunosuppression [11]. In clinical practice, newly presenting exuberant, explosive, erythrodermic, rupioid, treatment-refractory, or otherwise unusual psoriasis should therefore prompt consideration of HIV testing, while a positive result should lead to structured risk assessment rather than therapeutic nihilism [9,10,11,23,24].

Psoriasis severity may parallel uncontrolled HIV replication in some patients, and initiation or optimization of ART can improve psoriasis, particularly when disease activity coincides with high viral load or advanced immunodeficiency [9,10,11,23]. Conversely, immune reconstitution after ART may occasionally unmask or modify inflammatory dermatoses. HIV RNA and CD4 count should be interpreted as complementary but distinct markers: HIV RNA reflects virological control, whereas the CD4 count and its trajectory reflect immune reserve. Both should be considered alongside psoriasis severity and treatment urgency when systemic immunomodulation is contemplated [9,10,11,23,24]. When HBV or HCV is also present, this risk assessment becomes more complex because antiviral regimens, hepatic status, and immunosuppression must be considered jointly [12,13,18,19,20].

### 2.2. HIV and Viral Hepatitis Coinfection

In patients with HIV/HBV or HIV/HCV coinfection, clinical assessment should move beyond evaluation of each virus in isolation. Coinfection is clinically relevant because HIV can accelerate liver fibrosis, increase the risk of end-stage liver disease, complicate antiviral selection, and influence drug-interaction profiles [12,13,18,19,20]. Before systemic psoriasis therapy, clinicians should review, in practical sequence, the current ART regimen, whether it includes HBV-active agents, potential interactions with DAAs, renal function, liver fibrosis stage, and the degree of immunosuppression, ideally in collaboration with infectious disease and hepatology specialists.

A practical implication is that ART optimization may simultaneously improve HIV control and, when tenofovir- or lamivudine/emtricitabine-containing regimens are appropriate, contribute to HBV suppression. However, withdrawal or substitution of HBV-active ART in an HBV-coinfected patient can precipitate HBV flare. Dermatologists should therefore avoid making psoriasis-treatment decisions without confirming the current ART regimen and its HBV activity.

### 2.3. Psoriasis and HBV

HBV can persist in hepatocytes as covalently closed circular DNA (cccDNA) even after apparent clinical resolution [12,13,15]. This persistent intrahepatic reservoir explains why loss of immune control during immunosuppression can permit renewed HBV replication. Subsequent immune recovery may then trigger immune-mediated hepatocellular injury, with a clinical spectrum ranging from asymptomatic increases in HBV DNA to hepatitis flares, hepatic decompensation, and, rarely, fatal liver failure [12,13,14,15,16,17].

Reactivation risk is determined jointly by viral serostatus, baseline HBV DNA, HBeAg and anti-HBs status, liver fibrosis, and the type and intensity of immunomodulation [12,13,14,15,16,17,25]. HBsAg-positive patients carry the highest reactivation risk and generally require antiviral treatment or prophylaxis with a high genetic barrier nucleos(t)ide analogue before or at initiation of systemic immunomodulation [12,13,14,15]. HBsAg-negative/anti-HBc-positive patients have a lower but non-zero risk; within this group, absent anti-HBs, detectable HBV DNA, combination or higher-risk immunosuppression, and inability to ensure reliable monitoring increase concern [14,15,16,17,25]. Clinically, HBV serology should therefore be interpreted together with markers of viral replication and the planned psoriasis therapy rather than in isolation.

### 2.4. Psoriasis and HCV

Unlike HBV, HCV is less strongly associated with abrupt viral reactivation during immunomodulatory therapy; however, active HCV infection remains clinically important because chronic infection may lead to progressive fibrosis, cirrhosis, hepatocellular carcinoma, mixed cryoglobulinemia, baseline transaminase abnormalities, and overlapping metabolic or alcohol-related liver disease [18,19,20,21,22].

Because current DAA regimens are short, well tolerated, and highly effective, HCV eradication should be integrated into the psoriasis treatment plan whenever feasible [18,19,20]. Urgent psoriasis treatment should not be excessively delayed, particularly in erythrodermic, pustular, disabling, or arthritis-associated disease; in such cases, HCV evaluation and treatment may proceed in parallel with psoriasis therapy after fibrosis staging and drug-interaction review [18,19,20,21,22]. Thus, the practical issue in HCV is less one of classical reactivation prophylaxis than of integrating viral eradication, hepatic reserve, potential hepatotoxicity and interactions, and the urgency of psoriasis control.

## 3. Baseline Assessment Before Systemic Therapy

Before initiating systemic psoriasis therapy, baseline assessment should be systematic and should not depend solely on known infection history. Patients may be unaware of chronic HBV, resolved HBV, HCV, or HIV infection, and risk factors may not be volunteered. Screening is particularly important before biologics, methotrexate, cyclosporine, TYK2/JAK-pathway agents, and oral targeted therapies [2,3,4,12,13,14,15,18,19,20,21,22,23,26].

HIV assessment should include HIV antigen/antibody testing if status is unknown. If positive, document HIV RNA viral load, CD4 count and trajectory, current ART regimen, adherence, history of opportunistic infections, resistance history when relevant, and coinfections including HBV, HCV, tuberculosis, and syphilis [9,10,11,23,24].

HBV assessment should include HBsAg, total anti-HBc, and anti-HBs. If HBsAg or anti-HBc is positive, obtain HBV DNA and ALT/AST; if HBV DNA is detectable, management should be escalated to hepatology or infectious disease [12,13,14,15,16,17,26]. Susceptible patients should be vaccinated against HBV before or during psoriasis care whenever feasible [12,13].

HCV assessment should include anti-HCV antibody and, when positive, HCV RNA. Genotyping may be unnecessary for some pangenotypic regimens but should follow local DAA protocols. Fibrosis assessment by elastography or validated non-invasive scores is important before hepatotoxic therapy [18,19,20,21,22].

General liver assessment should include ALT, AST, alkaline phosphatase, gamma-glutamyl transferase, bilirubin, albumin, INR, platelet count, alcohol history, metabolic risk, ultrasound when indicated, and fibrosis staging. Vaccination history should include HAV vaccination in chronic liver disease or HCV, HBV vaccination in susceptible patients, influenza, pneumococcal, COVID-19, HPV, and recombinant zoster vaccination when appropriate. Live vaccines should generally be administered before substantial immunosuppression or avoided during it [2,3,4,12,13,18,19,20,23].

## 4. Treatment of Psoriasis in People Living with HIV

### 4.1. General Principles

Management begins with ART optimization and confirmation of virological control [9,10,11,23,24]. In mild disease, topical corticosteroids, vitamin D analogues, keratolytics, topical calcineurin inhibitors for face or folds, and localized approaches are appropriate [2,3]. In moderate-to-severe disease, NB-UVB phototherapy, acitretin, and apremilast remain valuable lower-immunosuppression options [9,10,11,23].

In controlled HIV infection, defined pragmatically by suppressed HIV RNA, stable or improving CD4 count, good ART adherence, no active opportunistic infection, and reliable follow-up, systemic psoriasis therapies should not be automatically withheld [23,24,27,28,29,30]. This is particularly relevant when psoriasis causes major functional impairment, erythroderma, pustulation, high DLQI, severe genital or palmoplantar involvement, or psoriatic arthritis.

### 4.2. Phototherapy and Non-Immunosuppressive Options

NB-UVB is often a useful first-line systemic-sparing option for moderate plaque psoriasis in HIV-positive patients. It has no direct interaction with ART and avoids hepatotoxicity. Limitations include access, adherence, cumulative UV exposure, and limited practicality in rapidly progressive, erythrodermic, or extensive disease [2,3,9,10,11,23]. PUVA may be effective but is less favored because of long-term carcinogenic risk and logistical burden [2,3].

Acitretin is non-immunosuppressive and may be useful in plaque, palmoplantar, pustular, or erythrodermic psoriasis associated with HIV [9,10,11,23]. It can be combined with phototherapy to reduce cumulative UV dose. However, acitretin has slower onset, limited monotherapy efficacy in some severe plaque phenotypes, teratogenicity, dyslipidemia, mucocutaneous adverse effects, and hepatotoxicity risk; these issues are amplified by metabolic syndrome, alcohol use, or viral hepatitis coinfection [3,18,19,20,21,22,23].

Apremilast is attractive when infection risk or drug monitoring is a major concern. It is not broadly immunosuppressive and has minimal laboratory monitoring requirements, although efficacy is generally lower than modern biologics and adverse effects include gastrointestinal symptoms, weight loss, and mood-related warnings [4,23]. It may be especially useful as a bridge while HIV, HBV, or HCV therapy is being optimized.

### 4.3. Methotrexate and Cyclosporine

Methotrexate and cyclosporine require caution in HIV. Methotrexate can cause myelosuppression, hepatotoxicity, pulmonary toxicity, and interactions, especially with trimethoprim-sulfamethoxazole, renal impairment, alcohol use, or viral hepatitis coinfection [3,23]. Cyclosporine is a potent immunosuppressant with nephrotoxicity, hypertension, and important interactions with protease inhibitors, cobicistat, azoles, macrolides, and selected antiviral regimens [3,23].

These drugs may still have a short-term rescue role in exceptional unstable psoriasis when the benefits outweigh the risks, but they are generally less attractive for long-term management in HIV-positive patients, particularly when viral hepatitis, renal disease, hypertension, or poor follow-up coexist [18,19,20,21,22,23].

### 4.4. Biologic Therapy in Controlled HIV

Biologics are no longer best framed as categorically contraindicated in HIV. Expert-panel guidance and real-world reports support cautious use in selected patients with controlled HIV infection [23,24,27,28,29,30]. Before initiation, confirm HIV RNA suppression, stable CD4 count, ART adherence, absence of active opportunistic infection, tuberculosis status, and vaccination plan. Follow-up should include dermatological response, infection events, HIV RNA, and CD4 count at intervals agreed with the HIV specialist [23,24,27,28,29].

Anti-TNF agents have the longest historical experience but the broadest infection signal, particularly tuberculosis and opportunistic infections [2,23]. Ustekinumab, IL-17 inhibitors, and IL-23 inhibitors have increasing case-based and cohort evidence in controlled HIV [24,27,28,29]. IL-17 blockade may increase mucocutaneous Candida susceptibility, while IL-23 inhibitors may offer strong psoriasis efficacy with a generally favorable infection profile in the general psoriasis population [2,4,23]. No class has definitive randomized trial superiority in HIV-positive psoriasis because these patients are usually excluded from pivotal trials.

Quantitative clinical evidence in HIV remains observational but is increasingly informative. In the largest dedicated real-world cohort, Xu et al. followed 36 people living with HIV who received biologic therapy for psoriasis for 12 months: PASI75 was achieved by 36/36 (100%), PASI90 by 24/36 (66.7%), and PASI100 by 13/36 (36.1%). Documented infections occurred in 10% of the psoriasis cohort versus 10.5% of 144 age-, sex-, and ART-matched HIV-positive controls, and no significant longitudinal change in HIV RNA, CD4 count, or CD4 proportion was detected [28]. A systematic review covering 19 articles and 24 reported cases from 2018 to 2024 found improvement of cutaneous psoriasis in all reported cases; adverse events were seldom reported and were managed without treatment interruption, although standardized drug-survival analyses were not available [29].

### 4.5. Icotrokinra and Other Targeted Oral Agents

Icotrokinra is a first-in-class oral macrocyclic peptide that selectively blocks IL-23 receptor signaling [5,6,7,8]. It is approved in the United States for moderate-to-severe plaque psoriasis in adults and pediatric patients aged 12 years or older weighing at least 40 kg who are candidates for systemic therapy or phototherapy, at a recommended dose of 200 mg orally once daily [5]. Clinical development has shown superiority to placebo at week 16 and superiority to deucravacitinib in head-to-head phase 3 studies, with durable responses and a favorable safety profile reported through 52 weeks [5,6,7,8].

For patients with HIV or viral hepatitis, icotrokinra should be positioned as a targeted systemic agent with theoretical advantages over broad immunosuppressants but with limited direct evidence in uncontrolled chronic viral infection. Its oral route, selective IL-23 pathway targeting, and minimal expected hepatic metabolism may be attractive, but baseline TB and vaccination review are still recommended; severe hepatic impairment was not studied in registration trials, and real-world viral safety data are not yet mature [5,6,7,8]. Therefore, it should not bypass the same HIV/HBV/HCV screening, specialist input, and monitoring required for other systemic targeted therapies.

Deucravacitinib and other TYK2/JAK-pathway agents require similar caution. Although deucravacitinib is more selective than conventional JAK inhibitors, patients with uncontrolled HIV, active HBV, active HCV with significant liver disease, or recent opportunistic infection are not ideal first candidates until infection control and monitoring plans are established [4,12,13,14,15,18,19,20,21,22,23].

## 5. Treatment of Psoriasis in Patients with HBV

### 5.1. HBV Serological Categories

HBV status should be interpreted before systemic immunomodulation. Susceptible patients are HBsAg-negative, anti-HBc-negative, and anti-HBs-negative and should receive HBV vaccination. Vaccinated patients are HBsAg-negative, anti-HBc-negative, and anti-HBs-positive and do not have latent HBV reactivation risk. Resolved natural infection is defined by HBsAg negativity and anti-HBc positivity, with or without anti-HBs; reactivation risk is low but not zero. Chronic HBV infection is defined by HBsAg positivity, with variable HBV DNA and ALT. Occult HBV infection is characterized by HBsAg negativity, anti-HBc positivity, and detectable HBV DNA and should be managed as higher risk [12,13,14,15,16,17].

### 5.2. HBsAg-Positive or HBV-DNA-Positive Patients

HBsAg-positive or HBV-DNA-positive patients should be referred to hepatology or infectious disease before systemic immunomodulation whenever possible. HBV DNA, ALT, fibrosis stage, HBeAg status when relevant, and hepatocellular carcinoma risk should be assessed [12,13]. Antiviral prophylaxis or treatment with a high genetic barrier agent, typically entecavir, tenofovir disoproxil fumarate, or tenofovir alafenamide, should be started before or at the initiation of immunosuppressive or biologic therapy [12,13,14,15].

During psoriasis therapy, ALT and HBV DNA are commonly monitored every 1–3 months according to HBV risk, agent used, and guideline preference [13,14,15]. Antiviral therapy should continue throughout immunomodulatory treatment and for a defined post-treatment period; longer or indefinite therapy may be required depending on HBV treatment indications and the duration of psoriasis therapy [12,13,14,15].

### 5.3. Resolved HBV Infection

Patients with HBsAg-negative/anti-HBc-positive serology require individualized risk assessment. Serial ALT, HBsAg, and/or HBV DNA monitoring may be sufficient for lower-risk regimens and reliable follow-up, whereas prophylaxis should be considered if baseline HBV DNA is detectable, anti-HBs is absent, multiple immunosuppressants are used, high-risk regimens are planned, or monitoring cannot be assured [14,15,16,17,25].

### 5.4. Treatment Selection in HBV

Topical therapy and NB-UVB are preferred for mild-to-moderate disease when feasible. Acitretin and apremilast generally have low HBV reactivation concern but still require liver and metabolic monitoring [3,23]. Methotrexate is often undesirable in chronic HBV because of hepatotoxicity and the difficulty of distinguishing drug-induced liver injury from viral hepatitis [3,12,13,14,15]. Cyclosporine and biologics can be used when necessary, but HBsAg-positive patients should receive antiviral prophylaxis or treatment [12,13,14,15,16,17,25].

TNF inhibitors and ustekinumab have documented HBV reactivation cases and should not be used without a clear prophylaxis and monitoring plan in at-risk patients [16,26,31]. Secukinumab and other IL-17 inhibitors appear feasible under structured monitoring, but HBV reactivation remains possible [32,33]. IL-23 inhibitors have limited but encouraging experience in chronic HBV; nevertheless, they should be managed according to standard HBV screening and prophylaxis principles rather than assumed to be risk-free [21,22,34]. Icotrokinra should follow the same principle until specific viral hepatitis safety data are available [5,6,7,8].

Quantitative estimates confirm that HBV risk is strongly modified by serostatus and antiviral prophylaxis. In a meta-analysis of 10 observational studies including 238 patients, the pooled HBV reactivation rate during biologic therapy was 1.8% (95% CI 0.0–5.6%); rates were 4.1% in HBsAg-positive and 0.2% in HBsAg-negative patients. Among patients for whom antiviral prophylaxis was indicated, reactivation occurred in 26.6% without prophylaxis versus 0% with antiviral treatment [17]. A more recent single-center study and meta-analysis reported pooled reactivation rates of 21.2% in HBsAg-positive patients receiving cytokine inhibitors without prophylaxis and 4.4% in HBsAg-negative/anti-HBc-positive patients [25].

Therapy-specific cohorts also illustrate both efficacy and residual viral risk. In a prospective multicenter cohort of 63 secukinumab-treated patients with HBV or HCV markers, mean PASI improvement was 76.1% in HBsAg-positive patients and 81.5% in HBsAg-negative/anti-HBc-positive patients. Among 46 HBV-infected patients who did not receive prophylaxis, 7 (15.2%) developed HBV reactivation; the rate was 24.0% in HBsAg-positive versus 4.17% in HBsAg-negative/anti-HBc-positive patients, whereas no reactivation occurred in patients receiving antiviral prophylaxis [32]. Evidence for IL-23 inhibitors remains much smaller: in a three-patient case series of chronic inactive HBV treated with guselkumab, risankizumab, or tildrakizumab plus entecavir, two patients achieved complete clearance and one improved from BSA 7%/PGA 3 to BSA 2%/PGA 2; no HBV reactivation occurred over a cumulative 173 weeks, although guselkumab was discontinued in one patient because of limited efficacy [34].

## 6. Treatment of Psoriasis in Patients with HCV

### 6.1. General Principles

All patients with a positive anti-HCV antibody should undergo HCV RNA testing. If HCV RNA is positive, referral for DAA therapy is recommended unless contraindicated [18,19,20]. HCV treatment should be part of the global psoriasis plan because viral eradication reduces hepatic uncertainty and may expand systemic psoriasis options.

### 6.2. Timing of Psoriasis Therapy and DAA Therapy

When psoriasis is stable, it is reasonable to treat HCV first and reassess psoriasis therapy after sustained virological response. When psoriasis is severe, disabling, erythrodermic, pustular, or complicated by psoriatic arthritis, psoriasis treatment may proceed in parallel with DAA therapy after interaction review [18,19,20,21,22,23]. Biologic agents have minimal pharmacokinetic interactions with DAAs, whereas cyclosporine and selected oral agents require closer interaction assessment.

### 6.3. Drug Selection in HCV

NB-UVB, topical therapy, apremilast, selected biologics, and carefully selected targeted oral agents are generally more attractive than hepatotoxic conventional systemic drugs in HCV-positive patients [18,19,20,21,22,23]. Methotrexate should be avoided in active hepatitis, significant fibrosis, cirrhosis, heavy alcohol use, or persistently abnormal liver enzymes [3,18,19,20,21,22,23]. Acitretin can worsen lipids and liver enzymes and should be used cautiously. Cyclosporine is not hepatotoxic in the same way as methotrexate, but renal, hypertensive, and interaction risks often limit its use [3,18,19,20,21,22,23].

The HCV-specific evidence is likewise mainly observational. In the systematic review by Snast et al., HCV reactivation occurred in 3/97 patients exposed to biologic therapy, corresponding to an estimated yearly rate of 2.42% [16]. In the prospective secukinumab cohort described above, mean PASI improvement among the 14 HCV-positive patients was 77.7%, while 1/14 (7.1%) developed enhanced HCV replication with hepatitis [32]. In a later multicenter retrospective cohort of 60 secukinumab-treated patients with HBV and/or HCV markers, 1/60 (1.7%) experienced combined HBV and HCV reactivation after 48 weeks; secukinumab was discontinued in that patient, whereas no other viral reactivation was observed during a mean treatment duration of 53.5 ± 37.5 weeks [33]. These estimates should not be interpreted as comparative class-specific risks because cohort composition, antiviral exposure, definitions of reactivation, and follow-up differed substantially across studies.

## 7. Treatment Class Considerations Across HIV, HBV, and HCV

Table 1 summarizes pragmatic class-level considerations. These are not absolute rules; treatment should be individualized according to psoriasis urgency, viral control, liver fibrosis, comorbidities, drug interactions, local labeling, and specialist advice.

The strength of evidence supporting treatment decisions is heterogeneous. Table 2 summarizes the design and size of key infection-specific studies together with quantitative psoriasis outcomes, viral events, adverse-event information, and treatment persistence/discontinuation when reported. Dedicated randomized trials in these populations and formal drug-survival analyses are generally lacking; therefore, percentages should be interpreted as descriptive estimates rather than head-to-head comparisons between drug classes.

## 8. Infection-Specific Practical Algorithms

**Figure 1:**
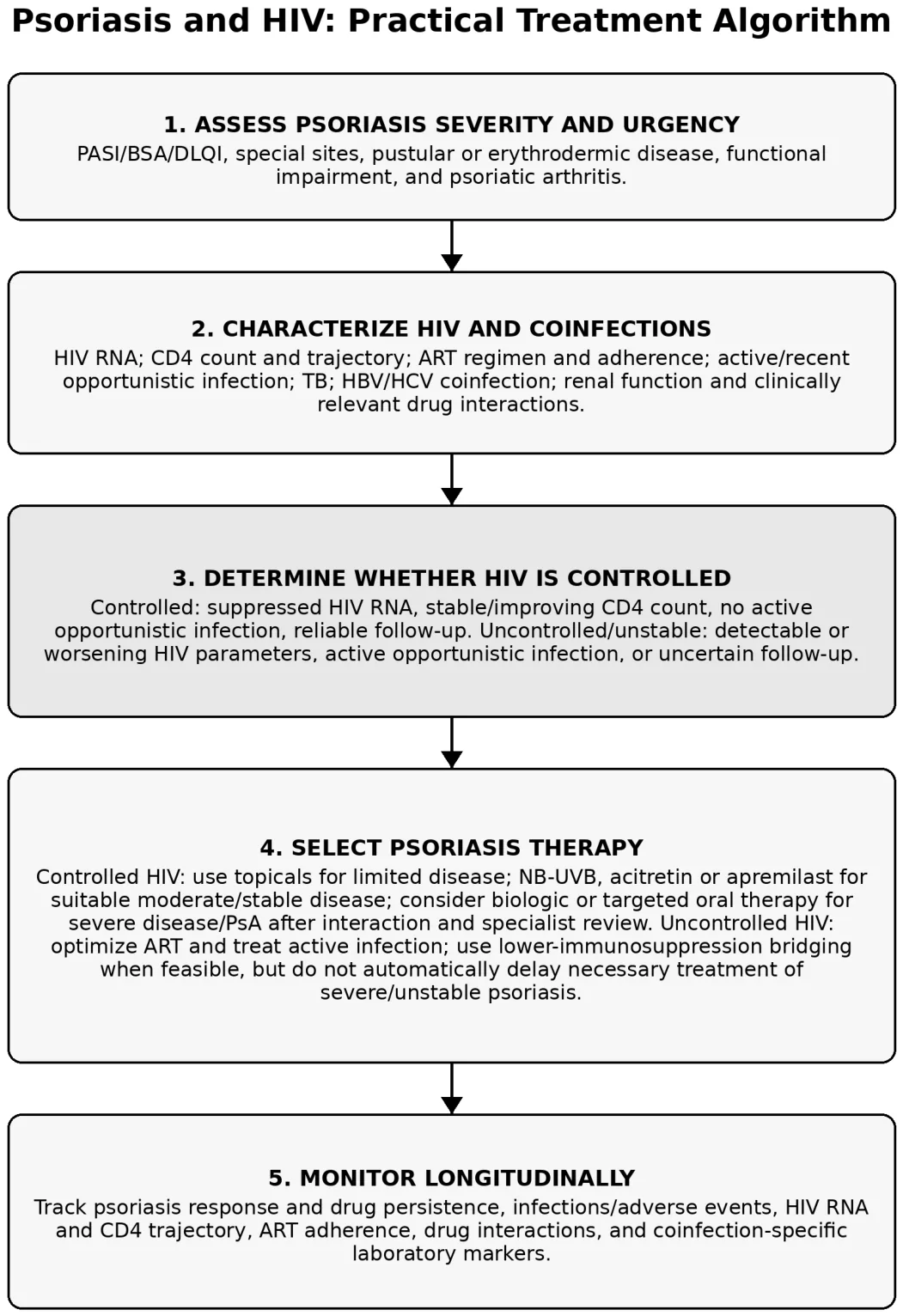
Infection-specific practical algorithm for psoriasis treatment in people living with HIV, integrating psoriasis urgency, HIV RNA, CD4 trajectory, ART, opportunistic infection status, treatment selection, and longitudinal monitoring.

**Figure 2:**
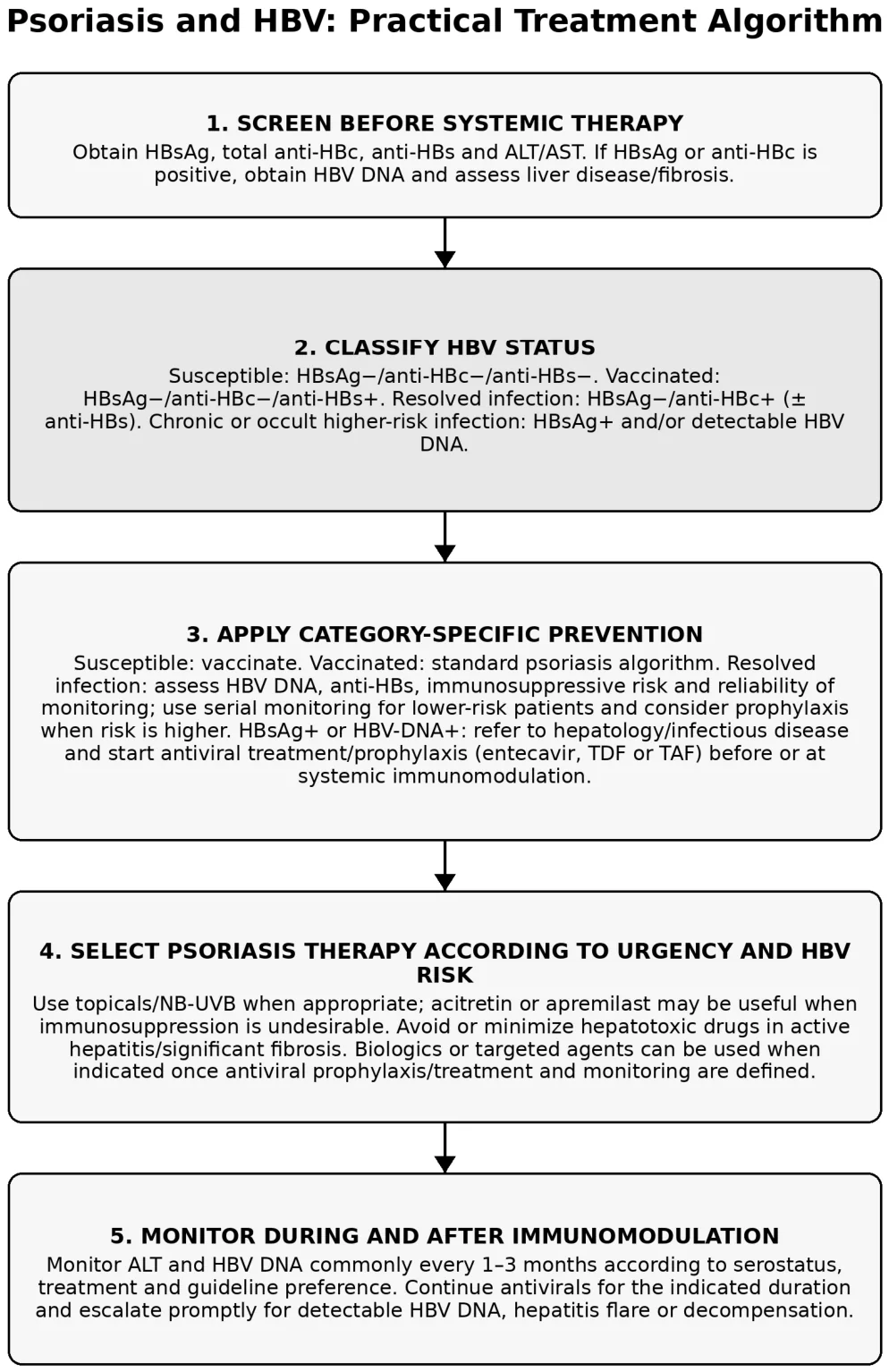
Infection-specific practical algorithm for psoriasis treatment in patients with HBV, stratified by HBsAg, anti-HBc, anti-HBs, HBV DNA, need for antiviral prophylaxis/treatment, liver disease, and longitudinal monitoring.

**Figure 3:**
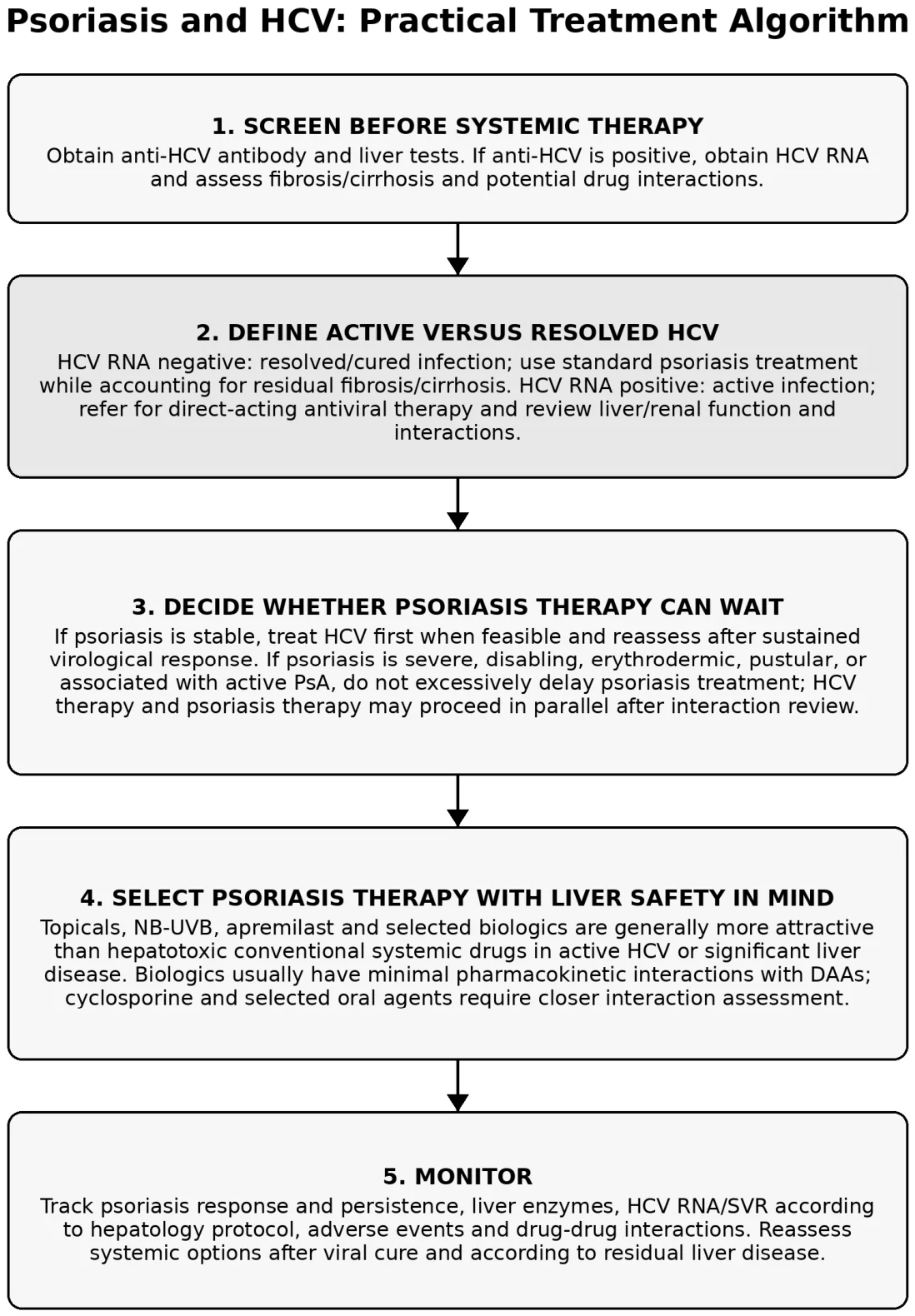
Infection-specific practical algorithm for psoriasis treatment in patients with HCV, distinguishing active from resolved infection and integrating fibrosis assessment, direct-acting antiviral therapy, drug-interaction review, treatment urgency, and follow-up.

Because HIV, HBV, and HCV modify systemic-treatment decisions through different mechanisms, the former combined pathway has been replaced by three infection-specific algorithms. Figure 1 addresses HIV according to virological control, immune reserve, opportunistic infection status, ART, and treatment urgency; Figure 2 stratifies HBV according to serology, HBV DNA, prophylaxis, and reactivation risk; and Figure 3 distinguishes active from resolved HCV and integrates fibrosis, DAA therapy, drug interactions, and the urgency of psoriasis control. These pathways are intended as pragmatic clinical frameworks rather than rigid treatment hierarchies [12,13,14,15,18,19,20,21,22,23,26].

## 9. Future Directions

The most important evidence gap is the near-systematic exclusion of patients with uncontrolled HIV, active HBV, active HCV, or advanced liver disease from pivotal psoriasis trials. This limits class-specific conclusions, particularly for IL-23 inhibitors, IL-17 inhibitors, deucravacitinib, and icotrokinra. Prospective registries should capture baseline viral status, ART and antiviral regimens, fibrosis stage, HIV RNA/CD4 trajectories, HBV DNA, HCV RNA, psoriasis outcomes, drug survival, serious infection, malignancy, and reactivation events [5,6,7,8,17,22,24,27,28,29,30].

Future work should also differentiate controlled infection from uncontrolled infection. Combining all HIV-positive or hepatitis-positive patients into a single risk category obscures clinically decisive differences between suppressed HIV with immune reconstitution and uncontrolled HIV with opportunistic infection; between vaccinated, resolved, occult, and chronic HBV; and between cured HCV and active HCV with cirrhosis.

The quantitative data summarized in Table 2 also demonstrate an important reporting limitation: standardized PASI endpoints, adverse-event denominators, treatment-discontinuation rates, and formal drug-survival analyses are inconsistently available, particularly for newer biologics and targeted oral agents. Future prospective cohorts should use harmonized definitions and report these outcomes alongside virological endpoints so that comparative benefit-risk estimates become possible.

## 10. Conclusions

HIV, HBV, and HCV infection should prompt structured assessment, not automatic exclusion from effective psoriasis treatment. In well-controlled HIV, systemic therapy, including selected biologics and targeted oral agents, may be considered with HIV-specific monitoring. In HBV, safe care depends on serological screening, HBV DNA assessment, antiviral prophylaxis or treatment in HBsAg-positive or HBV-DNA-positive patients, and serial monitoring. In HCV, DAA therapy should be integrated into the treatment plan, and hepatotoxic drugs should be avoided in significant fibrosis or cirrhosis.

Icotrokinra expands the therapeutic landscape by offering oral IL-23 receptor antagonism with high efficacy in moderate-to-severe plaque psoriasis, but its use in patients with chronic viral infection should remain risk-adapted until dedicated data are available. With screening, antiviral optimization, careful drug selection, and multidisciplinary follow-up, many patients with psoriasis and chronic viral infection can receive modern, effective, and safe care.

## Figures and Tables

**Table 1:** Pragmatic class-level considerations for psoriasis therapies in patients with HIV, HBV, or HCV infection.

Therapy/Class	HIV Considerations	HBV Considerations	HCV Considerations	Practical Implication
Topicals	Safe; first-line for limited disease.	Safe.	Safe.	Use in all patients as baseline therapy.
NB-UVB	Useful systemic-sparing option; no ART interaction.	No reactivation concern.	No reactivation concern.	Good option for moderate plaque disease when access and adherence allow.
Acitretin	Non-immunosuppressive; useful in palmoplantar, pustular, erythrodermic phenotypes.	Low reactivation concern; monitor liver and lipids.	Use cautiously; avoid significant liver disease or uncontrolled dyslipidemia.	Useful when immunosuppression is undesirable.
Apremilast	Favorable infection profile; modest efficacy.	Low reactivation concern.	Usually suitable; check interactions and tolerability.	Bridge or moderate-efficacy option.
Methotrexate	Caution: myelosuppression, infection, interactions.	Avoid or use very cautiously in chronic HBV because of hepatotoxicity.	Avoid in active hepatitis, fibrosis, cirrhosis, or alcohol excess.	Generally not preferred in viral hepatitis.
Cyclosporine	Potent immunosuppression; multiple ART interactions.	Reactivation risk; prophylaxis/treatment required if HBsAg-positive.	Renal/BP and interaction concerns.	Short-term rescue only in selected cases.
TNF inhibitors	Use only in controlled HIV; TB/opportunistic infection caution.	Higher HBV reactivation concern; prophylaxis and monitoring required in at-risk patients.	Can be used with monitoring.	Requires robust infection-screening plan.
Ustekinumab	Case-based supportive data in controlled HIV.	HBV reactivation reported; monitor or prophylax according to risk.	Generally feasible with monitoring.	Consider when phenotype/comorbidities fit.
IL-17 inhibitors	Increasing HIV case experience; monitor Candida and infections.	Reactivation possible; not risk-free.	Feasible with monitoring.	Potent option with structured follow-up.
IL-23 inhibitors	Limited but reassuring controlled-HIV data.	Limited data; reactivation uncommon but possible.	Attractive efficacy/safety profile with monitoring.	Useful after viral control, especially when avoiding broad immunosuppression.
Icotrokinra	No mature direct data in uncontrolled HIV; treat as targeted systemic therapy.	No mature HBV data; screen, vaccinate, and prophylax/monitor as indicated.	Minimal expected hepatic metabolism, but severe hepatic impairment not studied.	Potential oral targeted option after infection review; do not bypass viral-risk algorithm.
TYK2/JAK-pathway agents	Limited data; infection caution, avoid uncontrolled infection.	Screen and monitor carefully; consider prophylaxis in HBV risk.	Monitor liver tests and interactions.	Not first choice in uncontrolled viral infection.

Abbreviations: ART, antiretroviral therapy; BP, blood pressure; HBV, hepatitis B virus; HCV, hepatitis C virus; HBsAg, hepatitis B surface antigen; HIV, human immunodeficiency virus; NB-UVB, narrowband ultraviolet B; TB, tuberculosis; TYK2, tyrosine kinase 2.

**Table 2:** Quantitative outcomes and level of evidence for selected studies of systemic psoriasis therapy in patients with HIV, HBV, or HCV infection.

Study	Infection	Evidence Design/Sample	Therapy	Psoriasis Effectiveness	Viral Safety/Adverse Events	Persistence/Discontinuation
Xu et al. [28]	HIV	Retrospective cohort; 36 treated + 144 matched controls; 12 months	Etanercept, adalimumab, secukinumab, ustekinumab, risankizumab	PASI75 36/36 (100%); PASI90 24/36 (66.7%); PASI100 13/36 (36.1%)	Infections 10% vs. 10.5% in controls; no significant longitudinal change in HIV RNA, CD4 count, or CD4 proportion	Formal drug survival/discontinuation not reported
Shimon & Romanelli [29]	HIV	Systematic review; 19 articles, 24 reported cases	Apremilast and multiple biologics	Cutaneous improvement in 24/24 (100%); PsA improvement reported in 2 cases	Adverse events seldom reported and managed without treatment interruption	Formal drug survival not reported
Snast et al. [16]	HBV/HCV	Retrospective cohort *n* = 30 + systematic review of 49 studies/312 patients	Biologics	Psoriasis response not pooled	Cohort: 0 reactivations over a mean of 4.85 ± 3.1 years. Review: 2/175 anti-HBc-positive, 8/40 chronic HBV, and 3/97 HCV reactivations; yearly rates 0.32%, 13.92%, and 2.42%, respectively	Drug survival/discontinuation not reported
Wang et al. [17]	HBV	Systematic review/meta-analysis; 10 observational studies, *n* = 238	Biologics	Not pooled	HBV reactivation 1.8%; HBsAg-positive 4.1%; HBsAg-negative 0.2%; indicated prophylaxis absent 26.6% vs. prophylaxis 0%	Drug survival/discontinuation not reported
Kuo et al. [25]	HBV	Retrospective cohort *n* = 73 + meta-analysis of 10 studies	Cytokine inhibitors	Not pooled	Cohort: 0/11 HBsAg-positive and 2/62 (3.2%) HBsAg-negative/anti-HBc-positive reactivated; one reactivation was fatal. Meta-analysis: 21.2% in HBsAg-positive patients without prophylaxis and 4.4% in HBsAg-negative/anti-HBc-positive patients	Drug survival not reported
Chiu et al. [31]	HBV/HCV	Observational cohort; *n* = 18 (14 HBV, 4 HCV)	Ustekinumab	Effectiveness was not the primary endpoint	HBV reactivation in 2/7 (29%) HBsAg-positive patients without prophylaxis; 0/3 occult HBV; 1 HCV patient with cirrhosis had HCV reactivation/recurrent HCC; no significant aminotransferase increase	Discontinuation/drug survival not reported
Chiu et al. [32]	HBV/HCV	Prospective multicenter cohort; *n* = 63 (49 HBV, 14 HCV)	Secukinumab	Mean PASI improvement: 76.1% (HBsAg+), 81.5% (HBsAg−/anti-HBc+), 77.7% (HCV)	Without prophylaxis: HBV reactivation 7/46 (15.2%); HBsAg+ 24.0% vs. HBsAg−/anti-HBc+ 4.17%; HCV reactivation 1/14 (7.1%); no reactivation with antiviral prophylaxis	None of the 8 reactivation/enhanced-replication cases discontinued secukinumab
Megna et al. [33]	HBV/HCV	Retrospective multicenter cohort; *n* = 60; mean 53.5 ± 37.5 weeks	Secukinumab	Response not a primary endpoint/not reported quantitatively	Combined HBV/HCV reactivation in 1/60 (1.7%); no other reactivation reported	Secukinumab discontinued in the reactivation case: 1/60 (1.7%)
Ch’en et al. [34]	HBV	Case series; *n* = 3; cumulative follow-up 173 weeks	Guselkumab, risankizumab, tildrakizumab + entecavir	BSA/PGA: 7%/3 → 2%/2; 6%/4 → 0%/0; 50%/4 → 0%/0	No HBV reactivation in 3/3	Guselkumab stopped for limited response in 1/3 (33.3%); no safety discontinuations
Li et al. [30]	HBV/HCV/HIV	Systematic review/meta-analysis; 14 reports, 1033 patients	Biologics	Efficacy not pooled	Pooled viral reactivation: overall 4%; HBV 4%; HCV 7%; HIV 12% (wide confidence intervals and heterogeneous evidence)	Drug survival/discontinuation not reported

Abbreviations: anti-HBc, antibody to hepatitis B core antigen; BSA, body surface area; HBV, hepatitis B virus; HBsAg, hepatitis B surface antigen; HCC, hepatocellular carcinoma; HCV, hepatitis C virus; HIV, human immunodeficiency virus; PASI, Psoriasis Area and Severity Index; PGA, Physician Global Assessment; PsA, psoriatic arthritis. NR/not reported indicates that the original publication did not provide a standardized quantitative estimate.

## Data Availability

No new data were created or analyzed in this study. Data sharing is not applicable to this article.

## Abbreviations

The following abbreviations are used in this manuscript:

ALT	Alanine aminotransferase
anti-HBc	Antibody to hepatitis B core antigen
anti-HBs	Antibody to hepatitis B surface antigen
ART	Antiretroviral therapy
AST	Aspartate aminotransferase
BSA	Body surface area
DAA	Direct-acting antiviral
DLQI	Dermatology Life Quality Index
HBeAg	Hepatitis B e antigen
HBsAg	Hepatitis B surface antigen
HBV	Hepatitis B virus
HCV	Hepatitis C virus
HIV	Human immunodeficiency virus
IL	Interleukin
INR	International normalized ratio
JAK	Janus kinase
NB-UVB	Narrowband ultraviolet B
PASI	Psoriasis Area and Severity Index
PUVA	Psoralen plus ultraviolet A
RNA	Ribonucleic acid
TB	Tuberculosis
TNF	Tumor necrosis factor
TYK2	Tyrosine kinase 2
